# Multistable states in a system of coupled phase oscillators with inertia

**DOI:** 10.1038/srep42178

**Published:** 2017-02-08

**Authors:** Di Yuan, Fang Lin, Limei Wang, Danyang Liu, Junzhong Yang, Yi Xiao

**Affiliations:** 1 School of Physics and Electrical Engineering, Anyang Normal University, Anyang 455000, People’s Republic of China; 2 School of Science, Beijing University of Posts and Telecommunications, Beijing 100876, People’s Republic of China; 3 School of Physics, Huazhong University of Science and Technology, Wuhan 430074, People’s Republic of China

## Abstract

We investigate the generalized Kuramoto model of globally coupled oscillators with inertia, in which oscillators with positive coupling strength are conformists and oscillators with negative coupling strength are contrarians. We consider the correlation between the coupling strengths of oscillators and the distributions of natural frequencies. Two different types of correlations are studied. It is shown that the model supports multistable synchronized states such as different types of travelling wave states, π state and another type of nonstationary state: an oscillating π state. The phase distribution oscillates in a confined region and the phase difference between conformists and contrarians oscillates around π periodically in the oscillating π state. The different types of travelling wave state may be characterized by the speed of travelling wave and the effective frequencies of oscillators. Finally, the bifurcation diagrams of the model in the parameter space are presented.

Synchronization phenomena in phase oscillator networks are usually addressed by considering the paradigmatic Kuramoto model since it was proposed by Kuramoto in 1975^1^. This model has been applied for understanding the collective behaviors in many fields, such as the synchronous flashing of groups of fireflies^2^, the coupling of oscillatory neurons in the suprachiasmatic nucleus of the brain governing circadian rhythms^3^, the rhythm of pacemaker cells of the heart, the interaction of cells containing oscillatory chemically reacting constituents^4^, applauding persons in a large audience^5^, phase synchronization in electrical power distribution networks^6^,^7^,^8^, and even some other systems^9^,^10^,^11^,^12^,^13^,^14^,^15^,^16^,^17^. Theoretically, the classical Kuramoto model with its generalizations turn out to be the paradigms for synchronization problem, which have inspired a lot of works because of both their simplicity for mathematical treatment and their relevance to practice^11^,^18^.

The original Kuramoto model comprises *N* phase oscillators that are globally coupled through the sine of their phase difference. Specifically, the system involves *N* interacting oscillators *i* = 1,2, …, *N*. The ith oscillator has its own natural frequency *ω*_*i*_ chosen from a given probability density *g*(*ω*) and is characterized by its phase *ϕ*_*i*_, which is a periodic variable of period 2π. One of the key assumptions in the Kuramoto model is that the mutual coupling strength *K* between oscillator and the mean field is positive. Positive coupling tends to pull the phases of the oscillators together, thus favoring synchrony. A natural generalization of Kuramoto model is to allow *K* to have either sign. Negative coupling, on the other hand, pushes the phases apart and thus favors a phase difference of π. When both types of coupling are present, the system can become frustrated. Positive and negative communications coexist in biological systems, some studies have evaluated the significance of these communications on the synchronization in neural networks that consist of excitatory and inhibitory neurons^19^, which interact positively and negatively with their neighboring neurons, respectively. Inhibitory interactions have been proposed to suppress undesired synchronization^20^ and to destabilize synchronized neural networks^21^. Some authors considered the local interaction among oscillators and found evidence of glassy behaviors when both positive and negative coupling strengths were allowed simultaneously^22^,^23^. Hong and Strogatz studied the situation in which the coupling strength is regarded as an oscillator’s ability reacting to the mean field individually^24^,^25^. In their works, both positive and negative coupling strengths are present in the population. Oscillators with positive *K* behave like conformists by tending to fall in line with prevailing rhythm in the population, whereas those with negative *K* are repelled by the rhythm and act like contrarians. They found a surprising time-dependent state, a travelling wave state in which the mean field oscillates at a frequency different from the population’s mean natural frequency and the phase difference between conformists and contrarians are locked at an angle away from π. Positive and negative interactions are also very common in social systems such as human society. For example, conformists positively interact with the neighbors, following the neighbors’ opinion unconditionally, whereas contrarians negatively interact, always rejecting the neighbors’ idea.

The modification of the Kuramoto model with an additional inertial term was firstly reported by Tanaka, Lichtenberg, and Oishi^26^. These researchers have been inspired in their model by a previous phase model developed by Ermentrout to mimic the synchronization mechanisms observed in the firefly Pteroptix malaccae^27^. These fireflies synchronize their flashing activity by entraining to the forcing frequency with almost zero phase lag, even for stimulating frequencies different from their own intrinsic flashing frequency. The main ingredient to allow for the adaptation of the flashing frequency to the forcing one is to include an inertial term in a standard phase model for synchronization. Furthermore, networks of coupled phase oscillators with inertia have been recently employed to investigate the self-synchronization in power grids^28^,^29^,^30^, as well as in disordered arrays of underdamped Josephson junctions^31^. Explosive synchronization has been reported for a complex system made up of phase oscillators with inertia, where the natural frequency of each oscillator is assumed to be proportional to the degree of its node^32^,^33^. Recently the dynamics of the Kuramoto model with an inertia term have also been well examined in many other studies^34^,^35^,^36^,^37^.

In this paper, we will investigate the dynamics and synchronization properties of the generalized Kuramoto model consisting of conformists and contrarians with an inertial term for fully coupled systems. Here we will consider the correlation between the coupling strengths of oscillators and the distributions of natural frequencies. In the following, we report our main results.

## Model

We start by considering the generalized Kuramoto model with inertia, in which the dynamics of phase oscillators are governed by the following equations


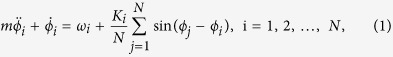


where *ϕ*_*i*_ is the phase of The *i* th oscillator at time *t, m* is the mass of an oscillator, *N* is the number of phase oscillators in the system. *ω*_*i*_ is the natural frequency of the *i*th oscillator and is chosen at random from a Lorentzian probability density *g*(*ω*) = *γ*/[*π*(*ω*^2^ + *γ*^2^)] of width *γ* and mean 〈*ω*〉 = 0 (the mean can be set to zero by choosing a suitable rotating frame). *K*_*i*_ is the coupling strength of the *i*th oscillator to the mean field, for simplicity, we assume that the coupling strength is chosen from a set comprising only two values: *K*_+_ > 0 and *K*_−_ < 0 represent the couplings for the conformists and contrarians, respectively.

The collective behavior in the model can be characterized by a mean field-like quantity, that is, a complex order parameter Re^*i*Φ^ which is defined as


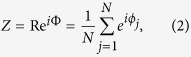


Here, *Z* is the average complex amplitude of all oscillators on the unit circle. *R* is the magnitude of complex amplitude characterizing the level of synchronization, and Φ is the phase of the mean-field corresponding to the peak of the distribution of phases.

In terms of *R* and Φ, substituting Eq. (2) into Eq. (1), we obtain the dynamical equation of the mean-field form


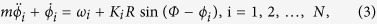


which expresses the evolution of the *i*th oscillator solely in terms of *R* and Φ. The complex order parameters in conformists and in contrarians are also important quantities to determine the dynamics in Eq. (1) and they are defined as 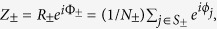 where *S*_±_ mean the set of conformists and contrarians, respectively, and *N*_±_ are the numbers of conformists and contrarians, respectively.

As for the correlation between the coupling strengths of oscillators and the distributions of natural frequencies, we consider two situations. In the first case, we let the coupling strength of the *i*th oscillator be *K*_*i*_ = *K*_+_ if |*ω*_*i*_| < *ω*_0_| and *K*_*i*_ = *K*_−_ otherwise, here, *ω*_0_ is defined as cut-off frequency. In the second case, we let the coupling strength of the *i*th oscillator be *K*_*i*_ = *K*_+_ if |*ω*_*i*_| > *ω*_0_| and *K*_*i*_ = *K*_−_ otherwise. The distributions corresponding to each case are as follows:


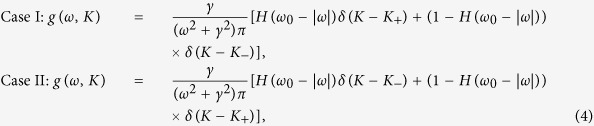


where H(·) is a Heaviside function. The two cases have their own motivations in reality. We take the first case as an example. In normal human society, people in the mainstream share similar opinions and they tend to be in harmony. On the other hand, there are extremists who may be in the right wing or left wing and they are prone to reject the opinions dominating the mainstream. To investigate the dynamics in such a human society the first situation might be a good candidate.

## Results and discussion

We numerically investigate the dynamics in Eq. (1) by a fourth-order Runge–Kutta algorithm with a time step δ*t* = 0.01 and the quantities of interest are measured after a sufficiently long transient is discarded. Throughout the work, we let *N* = 10000, *K*_−_ = −1.0, *K*_+_ = 3.0, m = 0.25 and *γ* = 0.05 unless specified. Initially, we assign each oscillator a phase randomly drawn from [0, 2*π*].

### A. Case with *K*_*i*_ = *K*_+_ for |*ω*_*i*_|<*ω*_0_ and *K*_*i*_ = *K*_−_ otherwise

**Figure 1:**
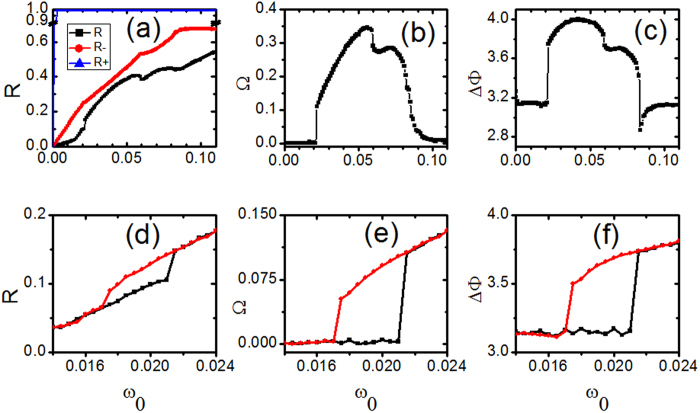
(**a**) The amplitudes of the order parameters R (the lower one), *R*_−_ (the middle one), and *R*_+_ (the upper one) against *ω*_0_. (**b**) The speed of a travelling wave Ω against *ω*_0_. (**c**) The phase difference ΔΦ between the average phases of the complex order parameters of contrarians *Z*_−_ and conformists *Z*_+_ against *ω*_0_. (**d**)–(**f**) The parameter region in (**a**)–(**c**) is zoomed in. In these three plots, two transition diagrams, labeled as forward (in black) and backward (in red) continuations, are presented. *K*_−_ = −1.0, *K*_+_ = 3.0, m = 0.25 and *γ* = 0.05.

Now, we investigate the case with *K*_*i*_ = *K*_+_ for |*ω*_*i*_| < *ω*_0_ and *K*_*i*_ = *K*_−_ otherwise. Firstly, we compute the synchronization diagrams of *R, R*_−_ and *R*_+_ against *ω*_0_, the results are presented in Fig. 1(a). When *ω*_0_ = 0 there are only contranrians. With the increase of *ω*_0_, the fraction of conformists increases and it is expected that the synchronization among oscillators becomes strong with *ω*_0_. We find that the coherence among conformists is very strong even for low *ω*_0_, so *R*_+_ almost stays around 1. On the other hand, both *R* and *R*_−_ show a strong dependence on *ω*_0_, the system presents several different regimes for different dynamical states. Several different regimes of *ω*_0_ can be separated by *ω*_0,1_ 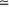 0.022, *ω*_0,1_ 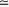 0.06 and *ω*_0,1_ 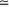 0.085, respectively.

These different regimes can be characterized through the speed of a travelling wave Ω, phase difference ΔΦ and phase distributions of oscillators. The quantity Ω is defined as 
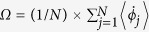
, which describes the velocity of phase oscillators moving as a whole along the phase space and Ω ≠ 0 refers to a travelling wave state. The quantity ΔΦ is defined as the phase difference between the average phases of conformists and contrarians, namely ΔΦ = Φ_+_ − Φ_−_). The speed of a travelling wave Ω against *ω*_0_ is presented in Fig. 1(b) in which Ω becomes nonzero in the range of *ω*_0_∈(*ω*_0,1_*ω*_0,3_). Correspondingly, Fig. 1(c) plotting the phase difference ΔΦ against *ω*_0_ shows that ΔΦ deviates from π in the same range. Both Fig. 1(b,c) demonstrate the existence of travelling wave state in the range of *ω*_0_∈(*ω*_0,1_ *ω*_0,3_). We note that the transition scenarios of *R* (Fig. 1(a)), Ω (Fig. 1(b)) and ΔΦ (Fig. 1(c)) in the forward continuation is not always continuous. In the range of *ω*_0_ < *ω*_0,1_ or *ω*_0_ > *ω*_0,3_, the *π* state is stable in which conformists and contrarians form two partially synchronized clusters and the peaks of their phase distributions are separated from each other by an angle of *π*. Ω and ΔΦ show sharp drops at around *ω*_0,2_, which indicate different types of traveling wave states on different sides of *ω*_0,2_.

Before going further, we have to point out that the transition at around *ω*_0,1_ is a discontinuous one. To demonstrate it, two types of transition diagrams, labeled as forward continuation and backward continuation, in the parameter range including *ω*_0,1_ are presented in Fig. 1(d,e,f). The forward continuation diagram is computed by increasing progressively the value of *ω*_0_ and the initial conditions for one *ω*_0_ are the final state of the previous one. The quantities *R*, Ω and ΔΦ are calculated for *ω*_0_,*ω*_0_ + δ*ω*_0_, …, *ω*_0_ + *n*δ*ω*_0_. Correspondingly, the backward continuation diagram is performed by decreasing progressively the value of *ω*_0_. The strong hysteresis displayed by the forward continuation diagram (in black symbols) and the backward continuation diagram (in red symbols) suggests a sharp and discontinuous transition at around *ω*_0,1_. The existence of hysteresis implies the coexistence of the cluster synchrony states and travelling wave states in a certain range of *ω*_0,1_.

**Figure 2:**
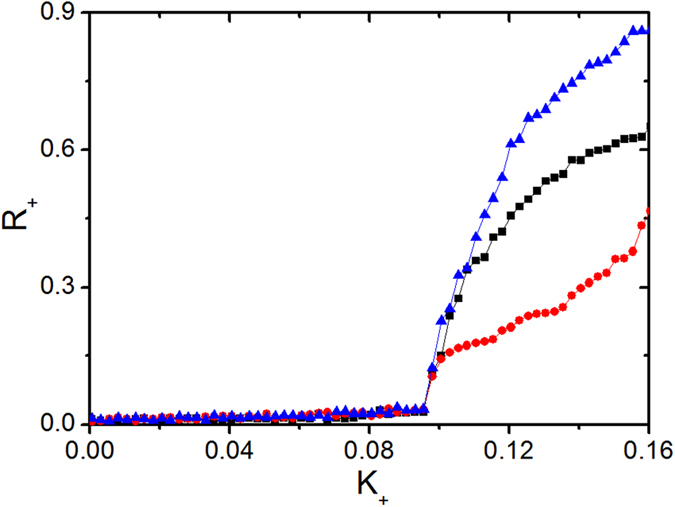
The amplitude of the order parameter *R*_+_ against *K*_+_ for different parameter sets: *K*_−_ = −1.0, *ω*_0_ = 0.4 in black (the middle curve); *K*_−_ = −1.0, *ω*_0_ = 0.1 in red (the lower curve); *K*_−_ = −1.0, *ω*_0_ = 0.1 in green (the upper curve).

Figure 1 also reveals that partially synchronized states are established once *ω*_0_ becomes nonzero. To understand it, we investigate the threshold of *K*_+_ for the onset of synchronization. The incoherence stability is determined by (see ref. 38)


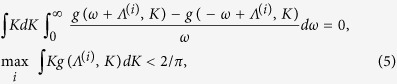


where Λ denotes the rotating frame frequency with respect to natural one, and in the second part of Eq. (5) we choose the maximum over possible solutions Λ^(*i*)^ of the first one. Due to the symmetry *g*(*ω, K*) = *g*(−*ω, K*), one of the solutions of Eq. (5) is always Λ^(*i*)^ = 0. Clearly, since *K*_−_ < *K*_+_ and the distribution of frequency is unimodal, the left hand of the second part of Eq. (5) achieves its maximum when Λ^(*i*)^ = 0, which is *K*_+_/*πγ*, so the incoherence looses its stability at *K*_+_,_C_ = 2. Thus, the incoherence stability is determined only by *K*_+_ and is not altered even if we set *K*_−_ → −∞, which is quite interesting. The claim is numerically confirmed in Fig. 2. Note that since the maximum of the second part of Eq. (5) is achieved at Λ^(*i*)^ = 0, the transition point coincides with the appearance of a stationary π state.

**Figure 3:**
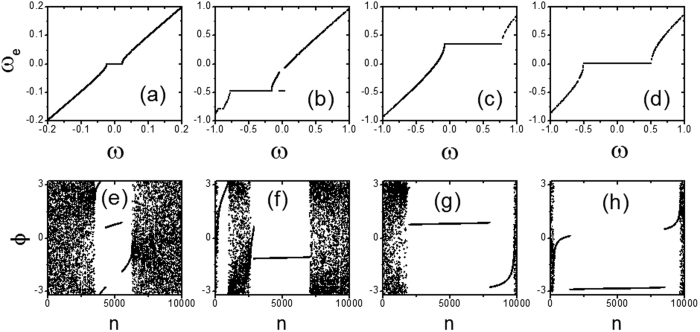
The effective frequencies of oscillators *ω*_*e*_ are plotted against their natural frequencies *ω* in (**a**), (**b**), (**c**) and (**d**). The snapshots of the phases of oscillators are showed in (**e**), (**f**), (**g**) and (**h**). The oscillators have been numbered according to their natural frequencies. (**a**,**e**) *ω*_0_ = 0.01; (**b**,**f**) *ω*_0_ = 0.04; (**c**,**g**) *ω*_0_ = 0.07; (**d**,**h**) *ω*_0_ = 0.1; *K*_−_ = −1.0, *K*_+_ = 3.0, m = 0.25 and *γ* = 0.05.

Next, we explore the dynamics of the system in different regimes in detail. We calculate the effective frequency *ω*_*e*_ for every oscillator and investigate the effective frequency as a function of the oscillators’ natural frequencies. The effective frequency of an oscillator is defined as *ω*_*e*_ = 〈*dϕ/dt*〉_*t*_ and the results are shown in the top panel in Fig. 3 for different *ω*_*0*_. At the same time, we plot the snapshots of the phases of oscillators in the bottom panel in Fig. 3 in which the oscillators have been numbered according to their natural frequencies (e.g., *i* < *j* if *ω*_*i*_ < *ω*_*j*_). For example, we take *ω*_*0*_ = 0.01 for *ω*_0_ < *ω*_0,1_ in Fig. 3(a–e), the system is in a stationary π state in which the graph *ω*_*e*_(*ω*) has one plateau for small |*ω*|. The oscillators on the plateau are phase-locked to the mean field and we have *ω*_*e*_ = 0 for them. The snapshot of the phases of oscillators shows that there are a small amount of contrarians which get synchronized. In addition, the plateau is connected and symmetric about *ω* = 0.

The graph of *ω*_*e*_(*ω*) becomes asymmetrical about *ω* = 0 for *ω*_0_ = 0.04 in the range of *ω*_0_∈(*ω*_0,1_ *ω*_0,2_), as shown in Fig. 3(b–f). The plateau is divided into two separated parts, one for conformists and the other for part of the contrarians. The effective frequency on the plateau deviates from 0. The observed asymmetry in the graph of *ω*_e_(*ω*) is related to the presence of traveling wave states. In the range of *ω*_0_∈(*ω*_0,2_ *ω*_0,3_), as shown in Fig. 3(c–g), the graph of *ω*_*e*_(*ω*) keeps asymmetrical about *ω* = 0 for *ω*_*0*_ = 0.07. However, the plateau is restored to be a connected one in the graph *ω*_*e*_(*ω*). At *ω*_*0*_ = 0.1 when *ω*_0_ > *ω*_0,3_, the plateau in the graph of *ω*_*e*_(*ω*)is back to a symmetrical one and π states are recovered [see Fig. 3(d–h)].

**Figure 4:**
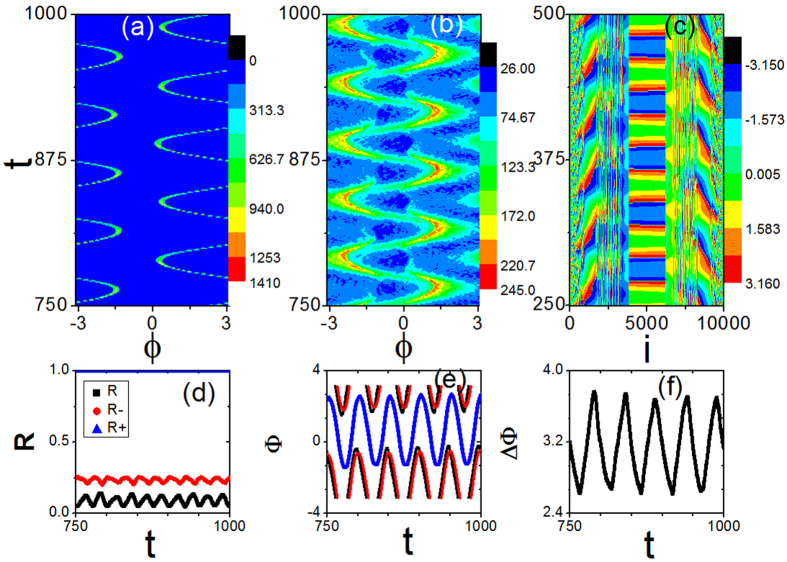
(**a, b**) The time evolutions of the phase distributions in the subpopulations of conformists and contrarians, respectively. (**c**) The time evolution of oscillators’ phases where the oscillators are numbered according to their natural frequencies. (**d**) The time sequences of the order parameters *R* (the lower one in black), *R*_−_ (the middle one in red), and *R*_+_ (the upper one in blue). (**e**) The evolutions of the average phase Φ (in black), Φ_−_ (in red), and Φ_+_ (in blue). (**f**) The evolutions of the phase difference ΔΦ. ΔΦ oscillates around *π* periodically, which refers to an oscillating *π* state. *ω*_0_ = 0.02, *K*_−_ = −1.0, *K*_+_ = 3.0, m = 0.25 and *γ* = 0.05.

Though several different dynamical states have been identified through the above investigations, there still exist other interesting states possessed in the system Eq. (1). One example is the states appearing when *ω*_0_ is close to *ω*_0,1_. In order to characterize the states, we consider the time evolutions of the phase distributions of the conformists in the Fig. 4(a) and the contrarians in the Fig. 4(b) for *ω*_0_ = 0.02. We know that the phase distributions of both conformists (P_+_(*ϕ*)) and contrarians ((P_−_(*ϕ*)) are stationary for a π state while they travel at the speed of a travelling wave Ω along the phase space for a travelling wave state. However, here Fig. 4(a,b) show a totally different state: the phase distributions do not travel through the phase space, and they are oscillating with a constant amplitude and a constant period. Furthermore, we investigate how conformists and contrarians organize themselves in an oscillating state. To do it, we number the oscillators according to their natural frequencies. If an oscillator *i* has its natural frequency *ω*_*i*_ and an oscillator *j* has the natural frequency *ω*_*j*_, then we have *i* < *j* if *ω*_*i*_ < *ω*_*j*_ regardless of their coupling strengths. The phase evolution of oscillators is shown in Fig. 4(c). Clearly, there exist several synchronous clusters in which adjacent oscillators stay closely in phase and the phase of each oscillator presents periodic change with time. Moreover, the period of the phase evolution of oscillators is the same as that of the evolutions of the phase distributions of both conformists and contrarians.

The periodic dynamics of the oscillating state can be manifested in the amplitudes of order parameters *R* and *R*_±_. Figure 4(d) shows the amplitudes of the order parameters *R* (the lower one in black), *R*_−_ (the middle one in red) and *R*_+_ (the upper one in blue) evolving with time and Fig. 4(e) shows the evolutions of the average phases Φ (the curve one in black), Φ_−_ (the curve one in red) and Φ_+_ (the curve one in blue). Moreover, Fig. 4(f) presents the phase difference ΔΦ between conformists and contrarians, which shows that ΔΦ oscillates around π with a constant period and a constant amplitude periodically. In this sense, we term the state as an oscillating π state.

Here we have to point out that the oscillating π state is different from the oscillating state in previous literatures. For example, Hansel *et al*.^39^ observed a slow periodic oscillation between two two-cluster states for the case of identical oscillators coupled through the first and the second Fourier modes. Bick *et al*.^40^ studied the coupled phase oscillator system with multi-harmonic couplings. In their study, it is shown that even symmetric systems of identical oscillators may exhibit chaotic mean field oscillations. If all conformists are synchronized, the periodic, quasiperiodic, and chaotic traveling waves are found recently in the subset of contrarians by Burylko *et al*.^41^. We investigate the model (1) and observe the oscillating π state. There are several typical characteristics for the oscillating π state. First, besides the amplitudes of the order parameters are oscillating, the average phases Φ and Φ_±_ are also oscillating periodically, and the phase difference ΔΦ oscillates around π with a constant period and a constant amplitude. Second, the phase distributions of both conformists and contrarians do not travel through the phase space, and they oscillate periodically in a confined region. Third, adjacent oscillators stay closely in phase and the phase of each oscillator presents periodic change with time. So, we think that the oscillating π state presented in our work may be complementary to the previous work.

Here there is an intriguing feature that should be addressed on oscillating π states. The amplitudes of the order parameters are oscillating at a half period of that of the phases. We consider the evolutions of the phase distributions of both conformists and contrarians in Fig. 4(a,b), the profiles of the phase distributions are time-dependent and the period they undergo is the same as that of Φ, Φ_+_, Φ_−_ and ΔΦ. However, Fig. 4(a,b) show that there are two moments in one period at which the phase distributions have the highest and narrowest peak. We know that the more concentrated the phases of the oscillators the more coherent the partially synchronized state, so the period of the amplitudes of the order parameters is half that of the phases.

**Figure 5:**
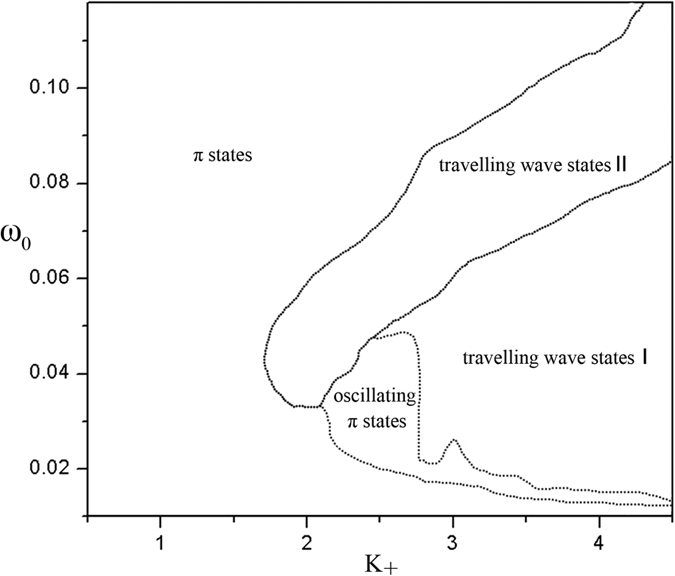
The bifurcation diagram of the model (1) on the space of *K*_+_ and *ω*_0_. The different curves divide the plane of *K*_+_ and *ω*_0_ into several regimes. Travelling wave states locate at strong *K*_+_ and the travelling wave state occurs in a wider window with the increase of *ω*_0_. Travelling wave states I is the travelling wave states in which the plateau in graph of *ω*_*e*_(*ω*) is divided into two pieces by desynchronized contrarians. Travelling wave states II is the travelling wave states in which the plateau in the graph of *ω*_*e*_(*ω*) is connected. The oscillating π states locate at the lower *ω*_0_ boundary of the travelling wave states. π states locate on the left sides of the travelling wave states and oscillating π states. *K*_*−*_ = −1.0, m = 0.25 and *γ* = 0.05. Here *K*_*i*_ = *K*_+_ for |*ω*_*i*_| < *ω*_0_ and *K*_*i*_ = *K*_−_ otherwise.

To get an overview on the dynamics in the model Eq. (1), we present the bifurcation diagram of the model Eq. (1) in the current correlation between the coupling strengths of oscillators and the distributions of natural frequencies in Fig. 5. Figuer 5 shows the parameter regimes for different dynamics, and different curves divide the plane of *K*_+_ and *ω*_0_ into several regimes. We find that the travelling wave states are prone to occur at strong *K*_+_ and the travelling wave state occurs in a wider window with the increase of *ω*_0_. We know that the travelling wave states can be classified into two types, one (travelling wave states I) in which the plateau in graph of *ω*_*e*_(*ω*) is divided into two pieces by desynchronized contrarians and the other (travelling wave states II) in which the plateau in the graph of *ω*_*e*_(*ω*) is connected. Travelling wave states IIare prone to occur at the upper *ω*_0_ boundary of travelling wave states I. We notice that the oscillating π states locate at the lower *ω*_0_ boundary of the travelling wave states. The π states can be found in the regime on the left sides of the travelling wave states and oscillating π states.

### B. Case with *K*_i_ = *K*_+_ for |*ω*_*i*_| > *ω*_0_ and *K*_i_ = *K*_−_ otherwise

**Figure 6:**
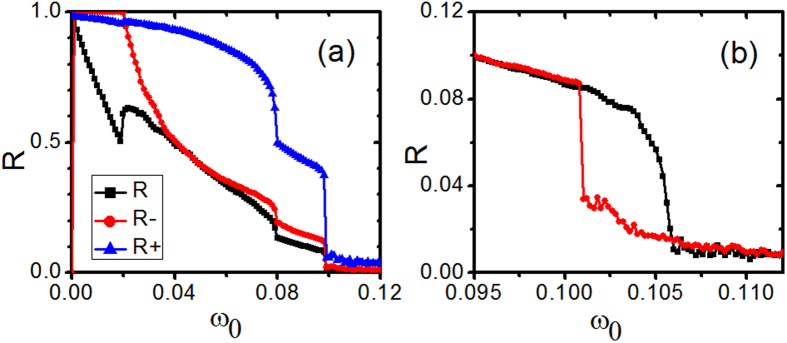
(**a**) The amplitudes of the order parameters *R*(the lower one), *R*_−_ (the middle one), and *R*_+_ (the upper one) against *ω*_0_. (**b**) The parameter region in (**a**) is zoomed in. In this plot, two transition diagrams, labeled as forward (in black) and backward (in red) continuations, are presented. *K*_−_ = −1.0, *K*_+_ = 3.0, m = 0.25 and *γ* = 0.05. Here *K*_*i*_ = *K*_+_ for |*ω*_*i*_| < *ω*_0_ and *K*_*i*_ = *K*_−_ otherwise.

Now, we investigate the case with *K*_i_ = *K*_+_ for |*ω*_*i*_| > *ω*_0_ and *K*_i_ = *K*_−_ otherwise. Different from the last situation, here contrarians have natural frequencies close to 〈*ω*〉 = 0, consequently, the fraction of conformists decreases with the increase of *ω*_0_. Firstly, we investigate the dynamics of the system and compute the synchronization diagrams of *R, R*_*−*_ and *R*_*+*_ against *ω*_0_ as shown in Fig. 6(a). The results show that there exist several regimes for different dynamical states. With the increase of *ω*_0_ from zero, the system encounters successively stationary π states, the travelling wave states in which the plateau in the graph of *ω*_*e*_(*ω*) is divided into two parts by desynchronized contrarians, the travelling waves states in which the plateau in the graph of *ω*_*e*_(*ω*) is a connected one, and the incoherent state. The transition between the travelling wave states and the incoherent state is a discontinuous one as shown in Fig. 6(b), we find the existence of hysteresis by the forward continuation diagram (in black symbols) and the backward continuation diagram (in red symbols). The existence of hysteresis implies the coexistence of the travelling wave states and the incoherent state at a certain range of *ω*_0_.

**Figure 7:**
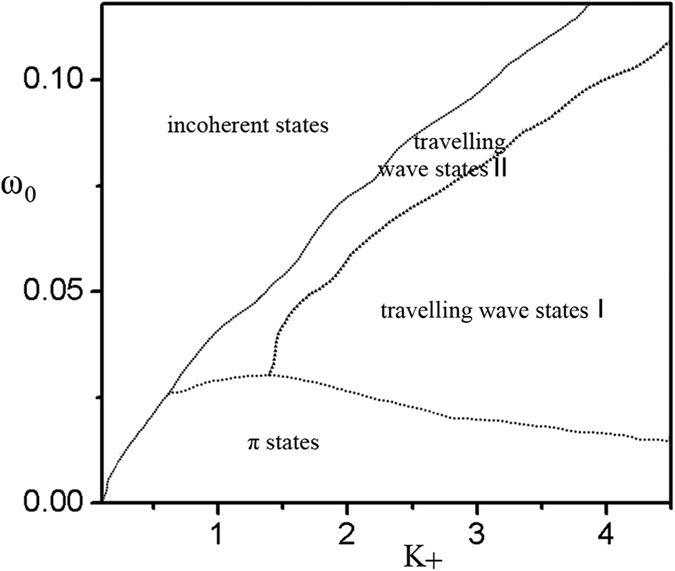
The bifurcation diagram of the model (1) on the space of *K*_+_ and *ω*_0_. The different curves divide the plane of *K*_+_ and *ω*_0_ into several regimes. Travelling wave states locate at strong *K*_+_ and at intermediate *ω*_0_. π states locate at the lower *ω*_0_ boundary of the travelling wave states which occurs at sufficient small *ω*_0_. The incoherent states locate on the left sides of the travelling wave states and π states. *K*_−_ = −1.0, m = 0.25 and *γ* = 0.05. Here *K*_*i*_ = *K*_+_ for |*ω*_*i*_| < *ω*_0_ and *K*_*i*_ = *K*_−_ otherwise.

To get an overview on the dynamics in the model Eq. (1) in the current correlation, the bifurcation diagram is presented in Fig. 7. Similar to the first case, we find that the travelling wave states are prone to occur at strong *K*_+_ and at intermediate *ω*_0_ as shown in Fig. 7. Travelling wave states II are prone to occur at the upper *ω*_0_ boundary of travelling wave states I. The π states locates at the lower *ω*_0_ boundary of the travelling wave states which occurs at sufficient small *ω*_0_. The incoherent states can be found on the left sides of the travelling wave states and π states.

## Conclusion

In this work, we consider the generalized Kuramoto model of globally coupled oscillators with inertia, in which oscillators with positive coupling strength are conformists and oscillators with negative coupling strength are contrarians. We introduce the correlation between the coupling strengths of oscillators and the distributions of natural frequencies into the model. Two different types of correlations are studied. The first case, conformists are those with natural frequencies close to the mean frequency of the population; the second case, contrarians are those with natural frequencies close to the mean frequency. We have studied the dynamics of the model in detail in different cases. We find that the model supports multistable synchronized states such as different types of travelling wave states, π state and, most interestingly, another type of nonstationary state, we termed it as an oscillating π state. The phase distribution oscillates in a confined region and the phase difference between conformists and contrarians oscillates around π periodically in oscillating π state.

Moreover, the travelling wave states can be classified into two types. The different types of travelling wave state may be characterized by the speed of a travelling wave Ω and the effective frequencies *ω*_*e*_ of oscillators. Furthermore, in the first case, the travelling wave may bifurcate from an oscillating π state at low *ω*_0_ or from a π state at high *ω*_0_. On the other hand, in the second case, the travelling wave seems to bifurcate continuously from a π state at low *ω*_0_ or discontinuously from the incoherent state at high *ω*_0_. Finally, the dynamics of the model in the parameter space are presented.

## Additional Information

**How to cite this article**: Yuan, D. *et al*. Multistable states in a system of coupled phase oscillators with inertia. *Sci. Rep.* **7**, 42178; doi: 10.1038/srep42178 (2017).

**Publisher's note:** Springer Nature remains neutral with regard to jurisdictional claims in published maps and institutional affiliations.
